# Supplementary material for: Site-Selective Solvation-Induced Conformational Switching of Heteroleptic Heteronuclear Tb(III) and Y(III) Trisphthalocyaninates for the Control of Their Magnetic Anisotropy
Source: Molecules. 2023 May 31;28(11):4474. doi: 10.3390/molecules28114474 (PMC10254442; doi:10.3390/molecules28114474)
Supplement: Supplementary file 1 [file molecules-28-04474-s001.zip › molecules-2397386-supplementary.pdf]

Site-selective solvation-induced conformational switching of heteroleptic heteronuclear Tb(III) and Y(III) trisphthalocyaninates for the control of their magnetic anisotropy

Alexander G. Martynov,[1,*] Kirill P. Birin,[1] Gayane A. Kirakosyan,[1,2] Yulia G. Gorbunova[1,2] and Aslan Yu. Tsivadze[1,2]

[1] Frumkin Institute of Physical Chemistry and Electrochemistry, Russian Academy of Sciences, Leninsky pr., 31, Building 4, 119071 Moscow, Russia; martynov@phyche.ac.ru (A.G.M.), kirill.birin@gmail.com (K.P.B.), gayakira@mail.ru (G.A.K.), tsiv@phyche.ac.ru (A.Yu.T.)
[2] Kurnakov Institute of General and Inorganic Chemistry, Russian Academy of Sciences, Leninsky pr., 31, 119991 Moscow, Russia; yulia@igic.ras.ru (Yu.G.G.)
* Correspondence: martynov@phyche.ac.ru (A.G.M.)

ELECTRONIC SUPPORTING INFORMATION

Figure S1. MALDI TOF mass-spectrum of **[B$_4$]Tb[B$_4$]Y[C$_4$].** 2

Figure S2. MALDI TOF mass-spectrum of **[B$_4$]Y[B$_4$]Tb[C$_4$].** 3

Figure S3. (a) Concentration-dependent UV-Vis spectra of **[B$_4$]Tb[B$_4$]Y[C$_4$]** in toluene; (b) Bouguer-Lambert-Beer plots of A/l vs. C for **[B$_4$]Tb[B$_4$]Y[C$_4$]** in toluene....................4

Figure S4. (a) Concentration-dependent UV-Vis spectra of **[B$_4$]Tb[B$_4$]Y[C$_4$]** in dichloromethane; (b) Bouguer-Lambert-Beer plots of A/l vs. C for **[B$_4$]Tb[B$_4$]Y[C$_4$]** in dichloromethane....................5

Figure S5. (a) Concentration-dependent UV-Vis spectra of **[B$_4$]Y[B$_4$]Tb[C$_4$]** in toluene; (b) Bouguer-Lambert-Beer plots of A/l vs. C for **[B$_4$]Y[B$_4$]Tb[C$_4$]** in toluene....................6

Figure S6. (a) Concentration-dependent UV-Vis spectra of **[B$_4$]Y[B$_4$]Tb[C$_4$]** in dichloromethane; (b) Bouguer-Lambert-Beer plots of A/l vs. C for **[B$_4$]Y[B$_4$]Tb[C$_4$]** in dichloromethane....................7

Figure S7. ^{1}H-^{1}H COSY of **[B$_4$]Tb[B$_4$]Y[C$_4$]** in toluene-*d*$_8$. 8

Figure S8. ^{1}H-^{1}H COSY of **[B$_4$]Tb[B$_4$]Y[C$_4$]** in CD_2Cl_2....................9

Figure S9. ^{1}H-^{1}H COSY of **[B$_4$]Y[B$_4$]Tb[C$_4$]** in toluene-*d*$_8$. 10

Figure S10. ^{1}H-^{1}H COSY of **[B$_4$]Y[B$_4$]Tb[C$_4$]** in CD_2Cl_2....................11

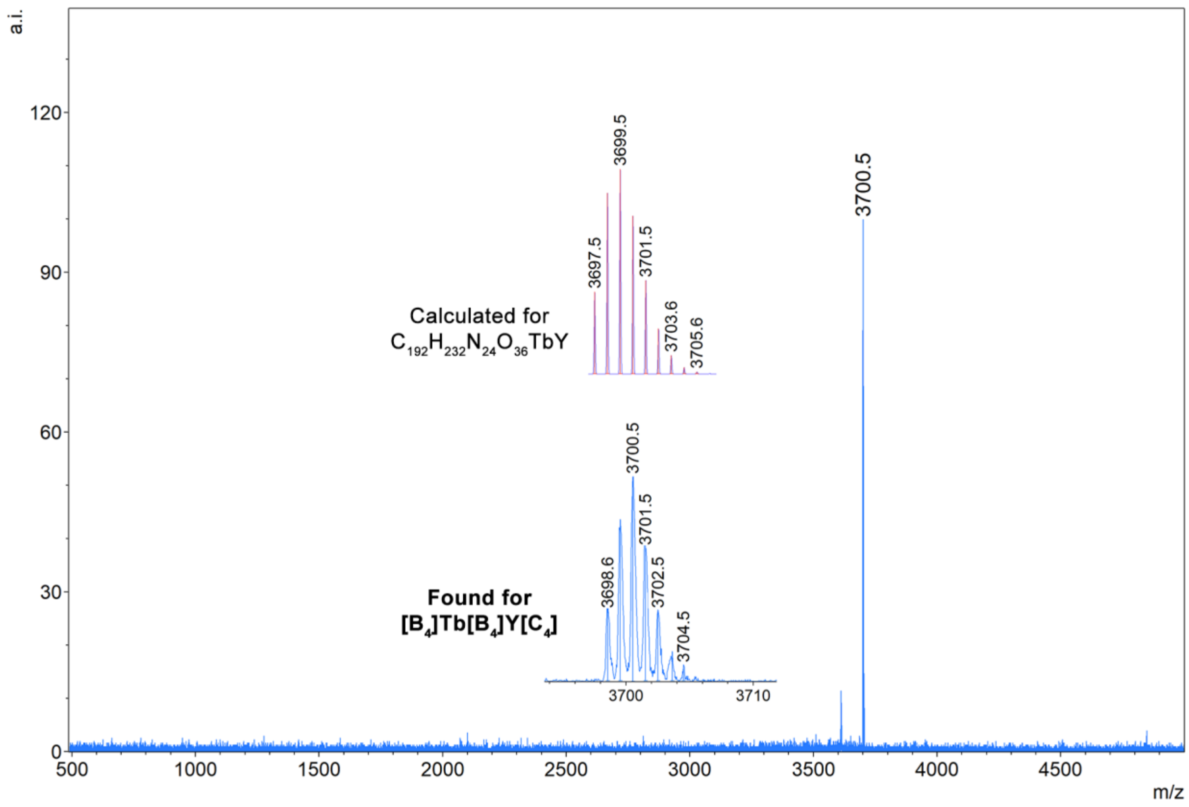

Figure S1. MALDI TOF mass-spectrum of **[B4]Tb[B4]Y[C4]**.

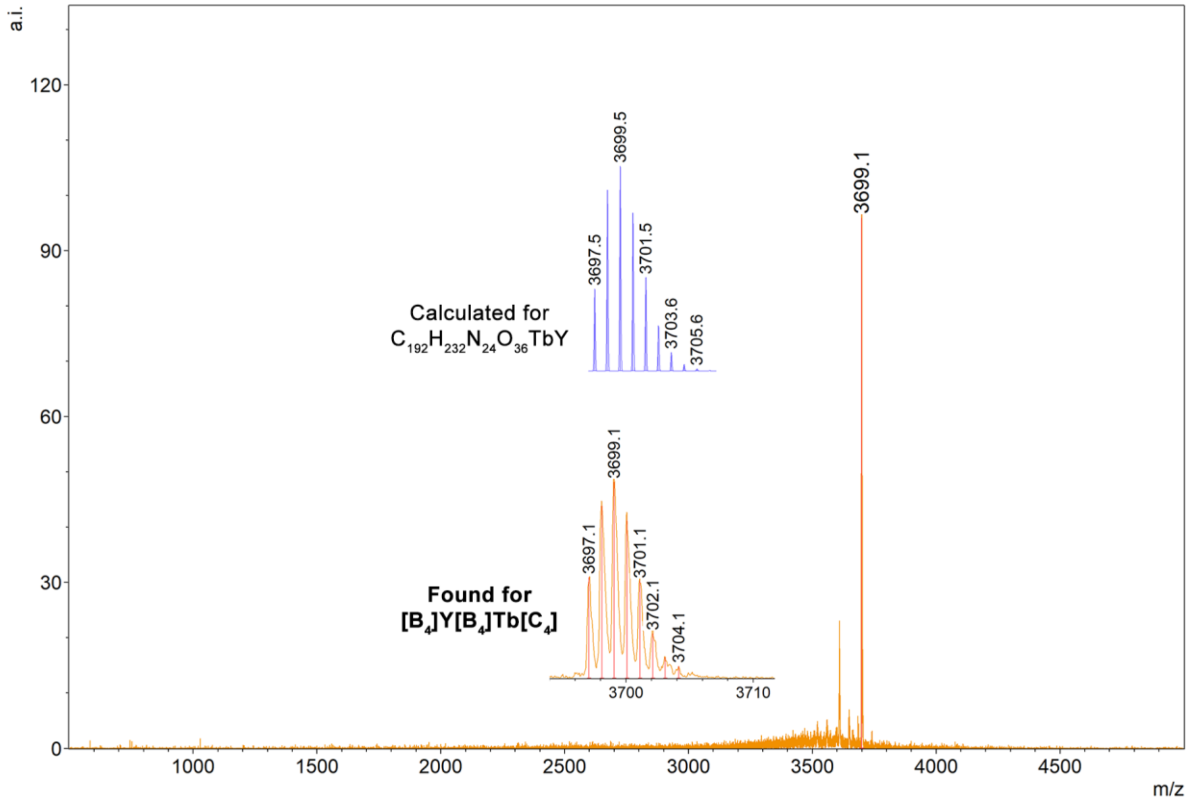

Figure S2. MALDI TOF mass-spectrum of **[B4]Y[B4]Tb[C4]**.

(a)

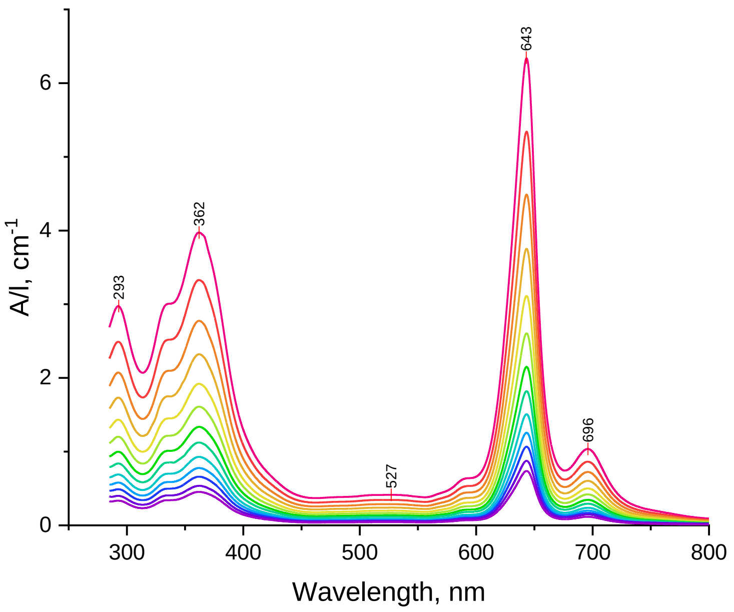

(b)

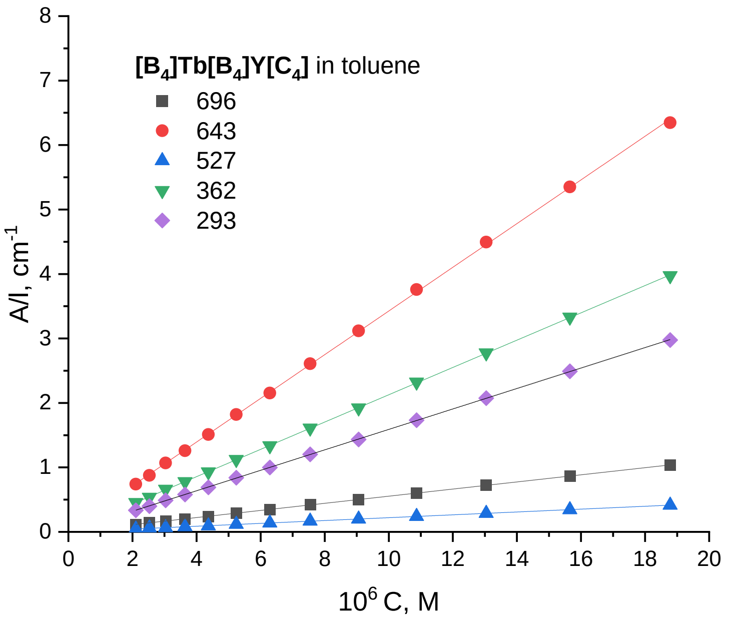

Figure S3. (a) Concentration-dependent UV-Vis spectra of **[B$_4$]Tb[B$_4$]Y[C$_4$]** in toluene; (b) Bouguer-Lambert-Beer plots of A/l vs. C for **[B$_4$]Tb[B$_4$]Y[C$_4$]** in toluene.

(a)

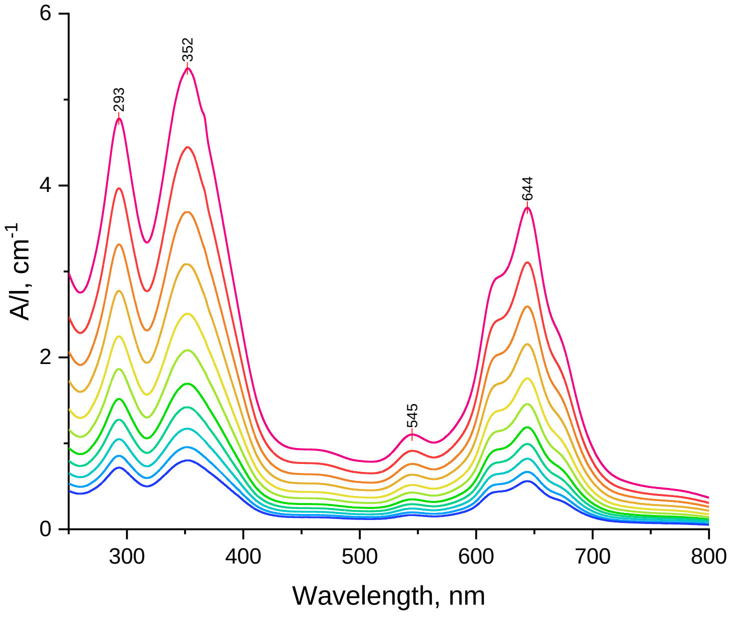

(b)

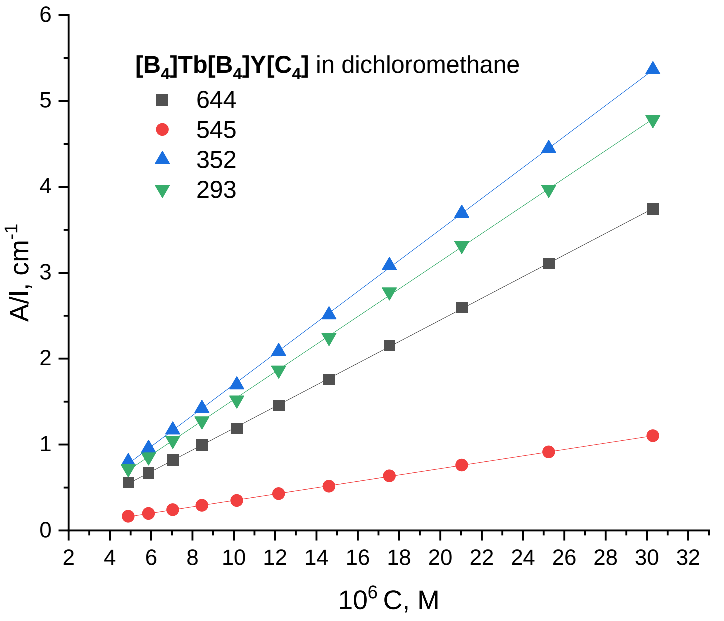

Figure S4. (a) Concentration-dependent UV-Vis spectra of **[B$_4$]Tb[B$_4$]Y[C$_4$]** in dichloromethane; (b) Bouguer-Lambert-Beer plots of A/l vs. C for **[B$_4$]Tb[B$_4$]Y[C$_4$]** in dichloromethane.

(a)

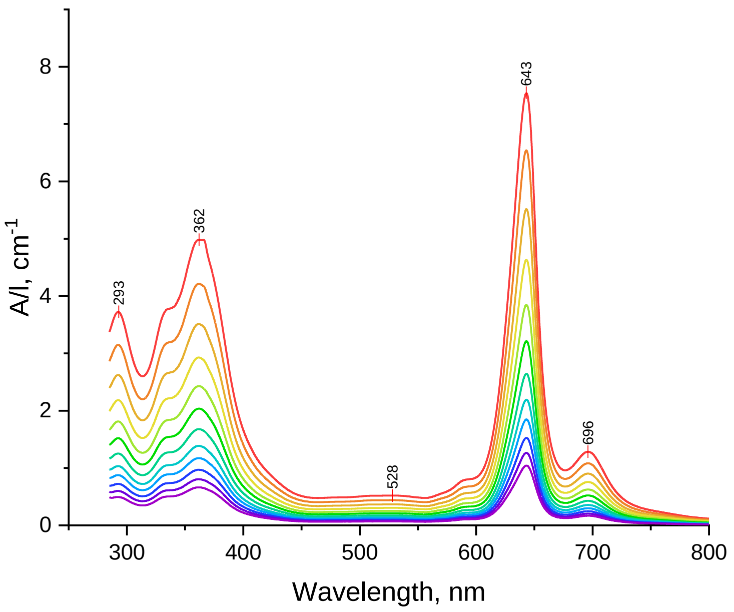

(b)

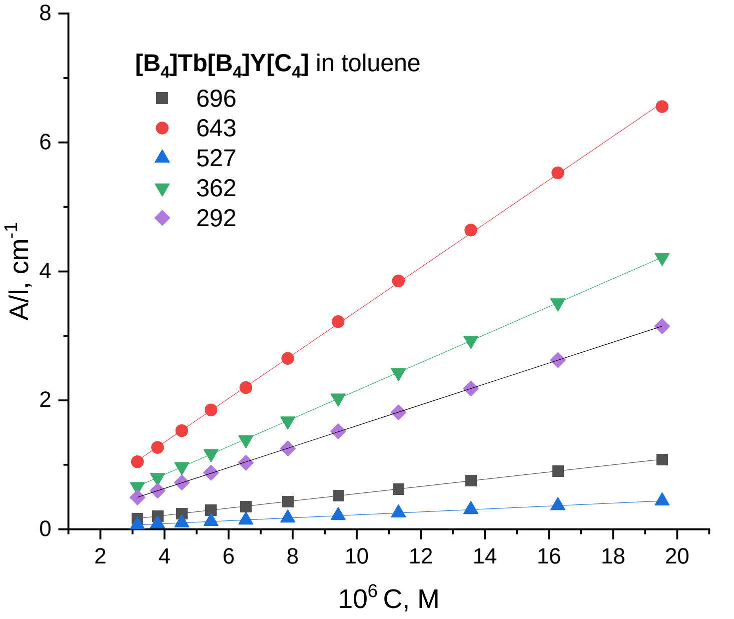

Figure S5. (a) Concentration-dependent UV-Vis spectra of **[B$_4$]Y[B$_4$]Tb[C$_4$]** in toluene; (b) Bouguer-Lambert-Beer plots of A/l vs. C for **[B$_4$]Y[B$_4$]Tb[C$_4$]** in toluene.

(a)

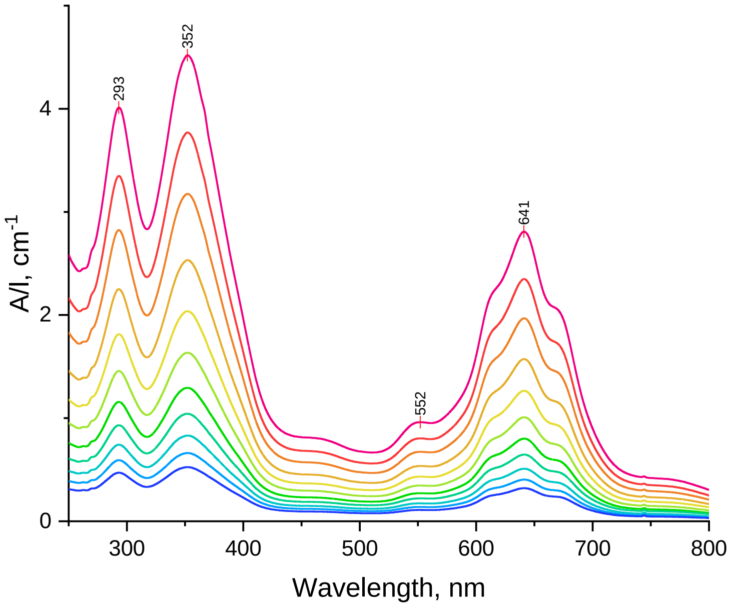

(b)

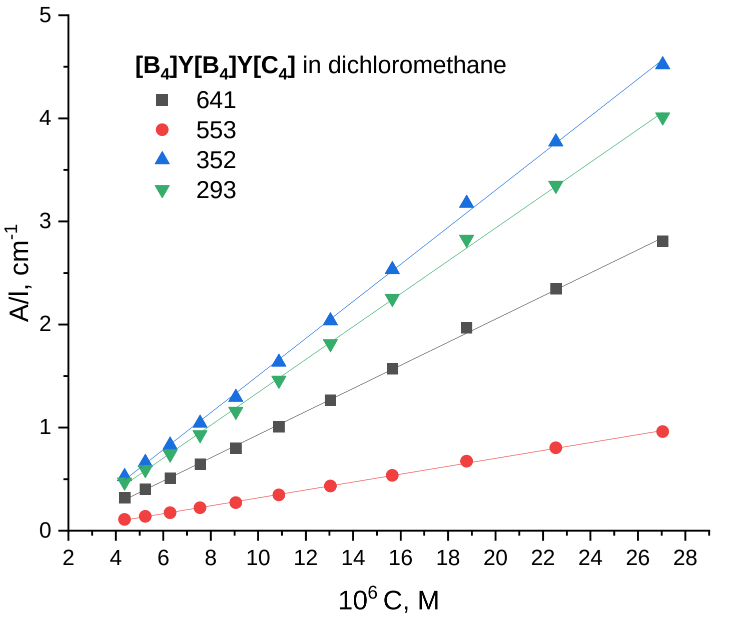

Figure S6. (a) Concentration-dependent UV-Vis spectra of **[B$_4$]Y[B$_4$]Tb[C$_4$]** in dichloromethane; (b) Bouguer-Lambert-Beer plots of A/l vs. C for **[B$_4$]Y[B$_4$]Tb[C$_4$]** in dichloromethane.

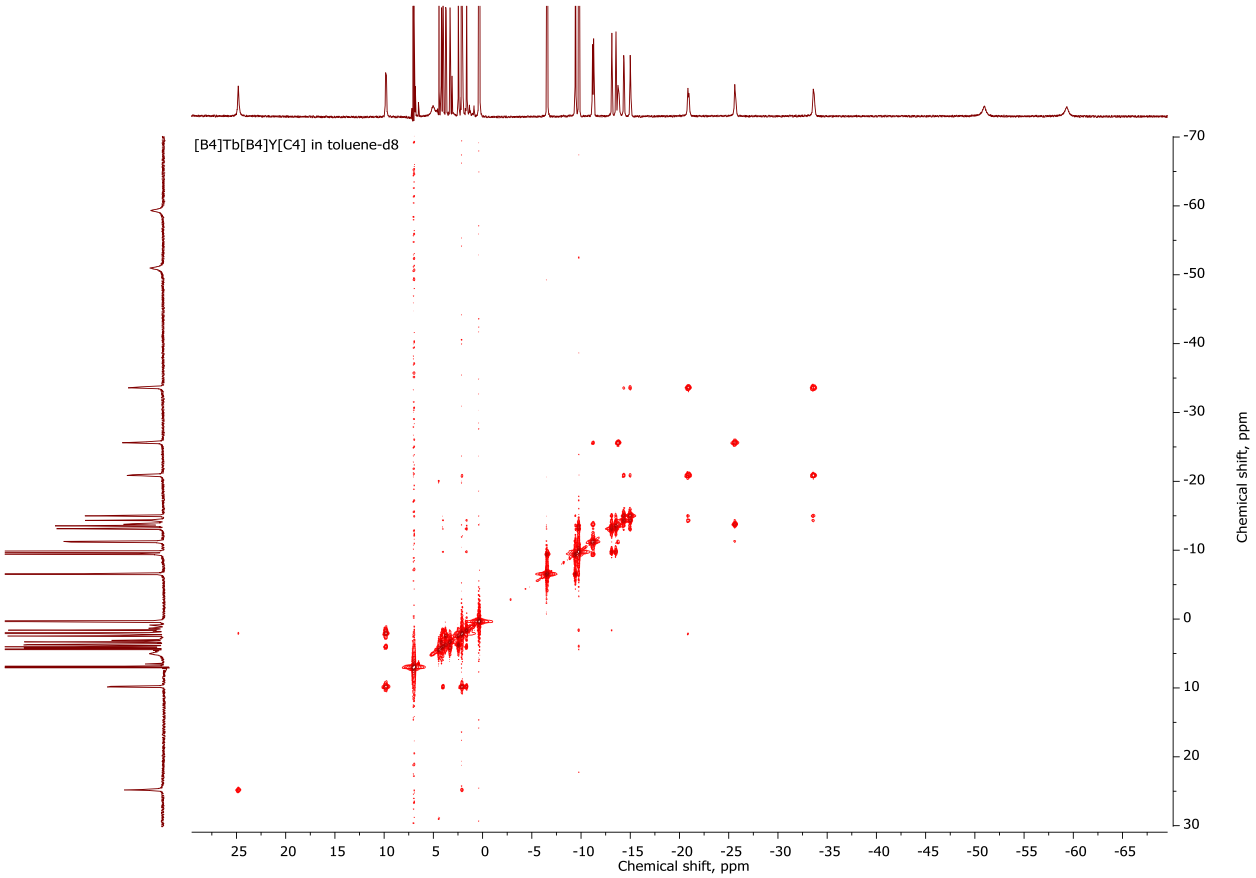

Figure S7. ^{1}H-^{1}H COSY of **[B$_4$]Tb[B$_4$]Y[C$_4$]** in toluene-d_8.

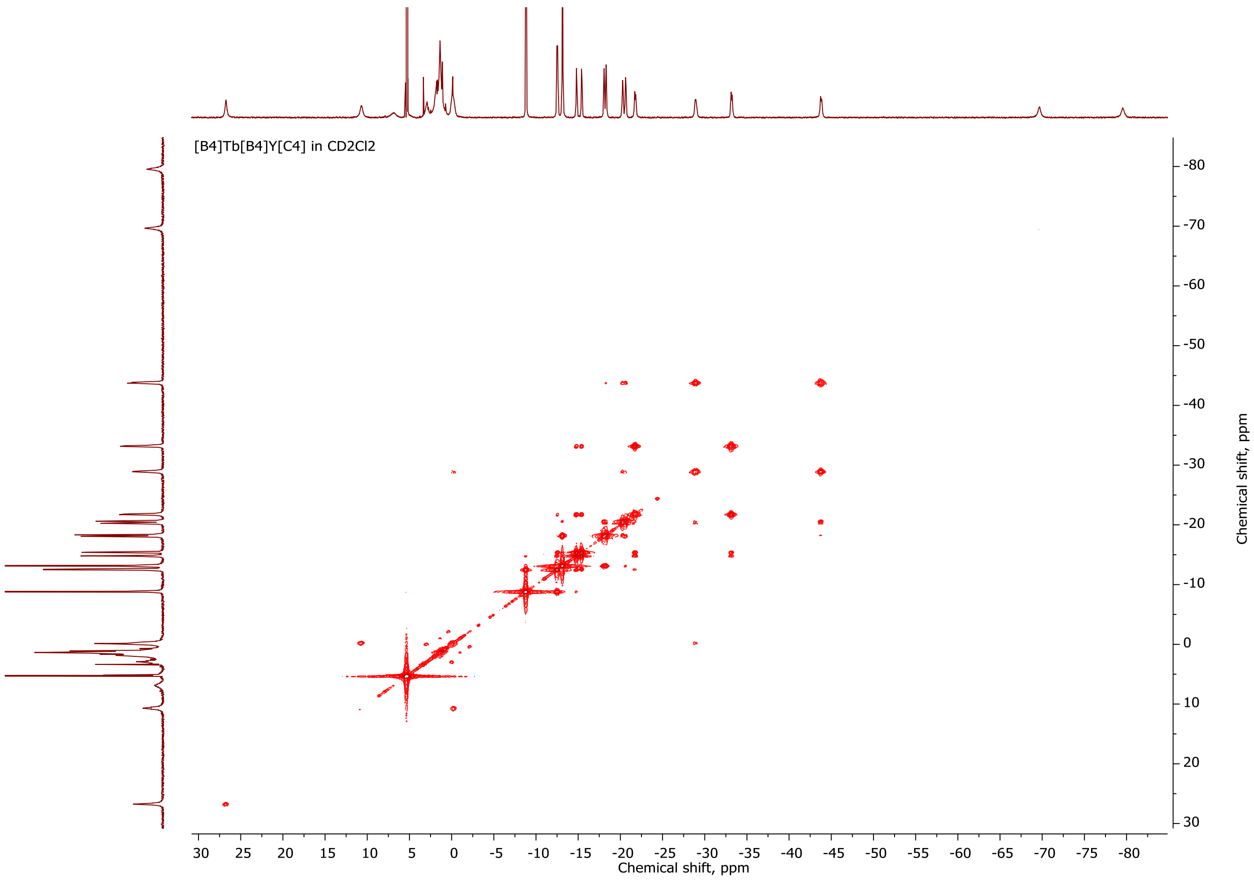

Figure S8. ^{1}H-^{1}H COSY of **[B$_4$]Tb[B$_4$]Y[C$_4$]** in CD_2Cl_2.

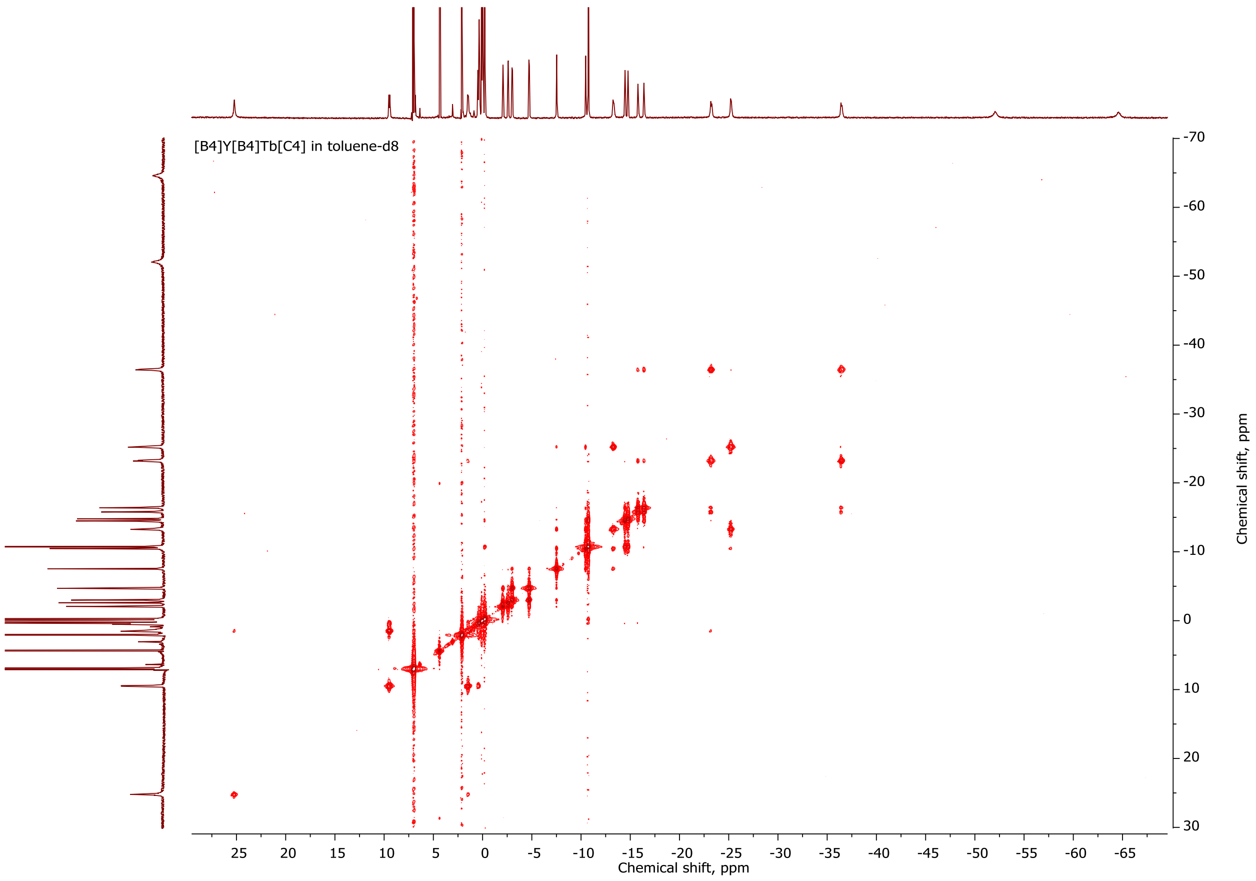

Figure S9. ^{1}H-^{1}H COSY of **[B$_4$]Y[B$_4$]Tb[C$_4$]** in toluene-d_8.

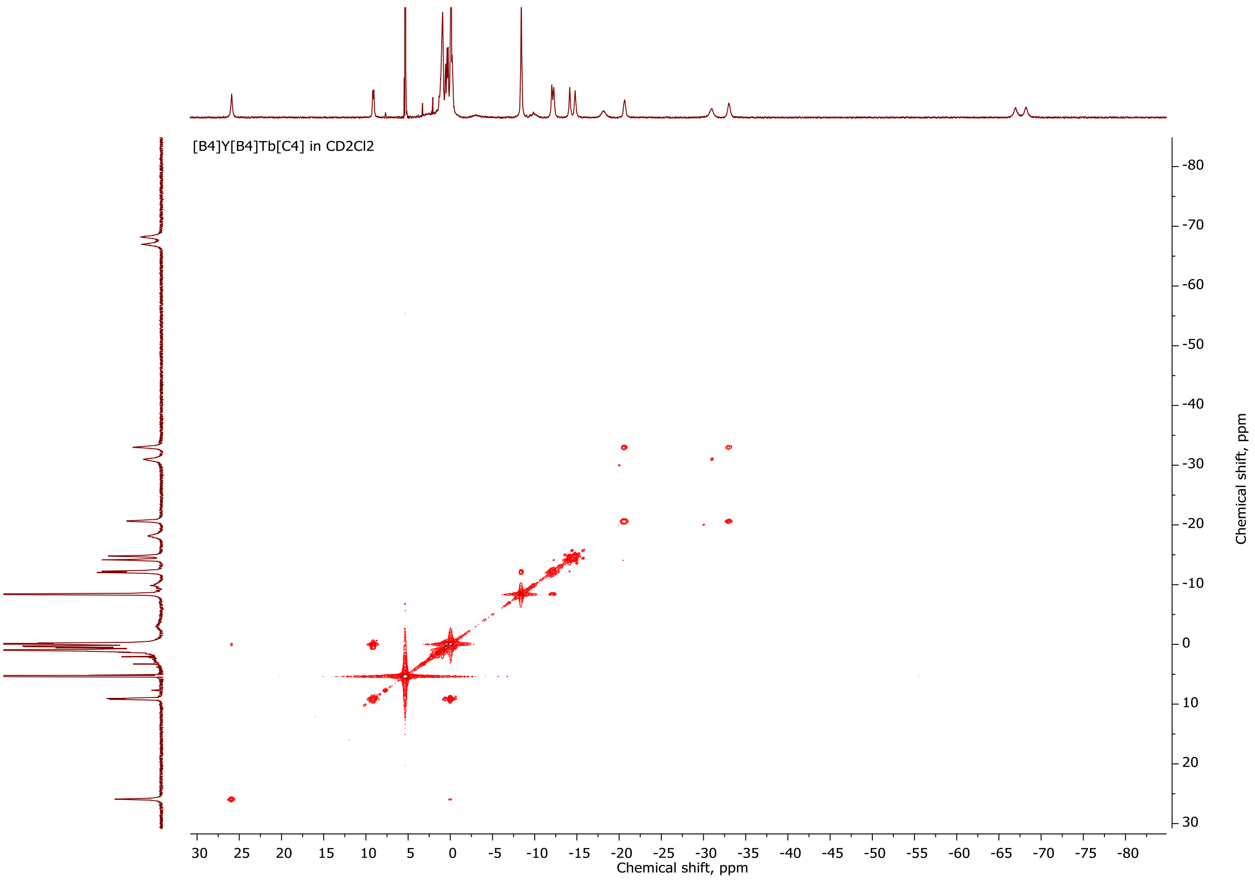

Figure S10. ^{1}H-^{1}H COSY of **[B$_4$]Y[B$_4$]Tb[C$_4$]** in CD_2Cl_2.
